# Dissecting the Simultaneous Extracellular/Intracellular Contributions to Cr(VI) Reduction under Aerobic and Anaerobic Conditions Using the Newly Isolating Cr(VI)-Reducing Bacterium of *Pseudomonas* sp. HGB10

**DOI:** 10.3390/microorganisms12101958

**Published:** 2024-09-27

**Authors:** Shenglei Chen, Xiaoyu Wang, Qinyi Zhao, Qiao Xu, Yini Zhang

**Affiliations:** School of Environment, Northeast Normal University, Changchun 130117, China; chensl008@nenu.edu.cn (S.C.); zhaoqy424@nenu.edu.cn (Q.Z.); xuq386@nenu.edu.cn (Q.X.); zhangyini@nenu.edu.cn (Y.Z.)

**Keywords:** Cr(VI)-reducing bacteria, MIC, reduction rate, extracellular space, intracellular component

## Abstract

Quantifying extracellular and intracellular contributions to Cr(VI) reduction is crucial for understanding bacterial Cr(VI)-reduction mechanisms. However, this contribution under different oxygen conditions remains largely unexplored. This study quantified the extracellular/intracellular contribution to aerobic and anaerobic Cr(VI) reduction using *Pseudomonas* sp. HGB10, an isolated Cr(VI)-reducing bacterium, as the experimental model. Interestingly, it was found that the lower anaerobic minimum inhibitory concentration (MIC) does not necessarily imply a lower anaerobic Cr(VI)-reduction rate for HGB10. For the initial Cr(VI) concentration of 20 mg L^−1^, the maximum anaerobic Cr(VI)-reducing rate reached 100%, while the aerobic counterpart was only 75%, even though the value of the aerobic MIC (400 mg L^−1^) is twice that of the anaerobic (200 mg L^−1^). Additionally, the calculated extracellular contributions to aerobic and anaerobic Cr(VI) reduction were 10.76% and 55.71%, respectively, while the intracellular contributions were 68.29% and 40.38%. The sum of extracellular and intracellular contributions to Cr(VI) reduction (79.05% and 96.09%) under aerobic and anaerobic conditions was nearly balanced with the corresponding maximum values despite minor relative errors. These results indicated that anaerobic Cr(VI) reduction mainly occurred extracellularly rather than intracellularly, which differs from the existing result. Overall, our findings provide new insights into bacterial Cr(VI) reduction.

## 1. Introduction

Oxygen conditions are essential to microbial growth, maintenance, and metabolism [1]. Molecular oxygen is typically not physicochemically homogeneous in natural aquatic and soil systems. Consequently, diverse microbial communities develop to adapt flexibly to oxygen gradients [2,3]. The same holds true for biological wastewater treatment systems, where oxygen heterogeneity, ranging from aerobic to microaerobic and anaerobic conditions, is also expected due to oxygen gradients in activated sludge or biofilm [4]. Therefore, understanding the microbial behaviors under various oxygen conditions is crucial to optimizing the oxygen operation conditions to achieve the desired degradation or removal of contaminants from wastewater.

Chromium is an essential material extensively used across various industries, including metallurgy, metal electroplating, leather tanning, and chemical processing [5,6]. The extensive application of chromium leads to substantial generation of hexavalent chromium-containing wastes, which pose significant environmental challenges. Microbial reduction of Cr(VI) offers a promising, cost-effective, and environmentally sustainable biological treatment approach to address these challenges [5]. This process, primarily executed by Cr(VI)-reducing bacteria, has garnered substantial attention due to its potential for effectively removing Cr(VI) from chromium-containing wastewater. These bacteria can employ multiple extracellular/intracellular mechanisms, such as biosorption, biotransformation, bioaccumulation, and bioreduction, to reduce toxic Cr(VI) to less toxic Cr(III) [6,7]. The bacterial Cr(VI)-reduction mechanism has become somewhat lucid. Nonetheless, simultaneous bacterial Cr(VI)-reduction studies in different oxygen conditions have been scarce. Most studies focus on investigating bacterial Cr(VI) reduction in aerobic or anaerobic conditions, partly because assessing aerobic and anaerobic extracellular/intracellular Cr(VI) reduction becomes more complex. For instance, *Shewanella oneidensis* MR-1 and *Escherichia coli*, among the most intensively studied Cr(VI)-reducing bacterial species, have not explored their Cr(VI)-reductive characteristics and mechanisms simultaneously under aerobic and anaerobic conditions [8,9,10,11,12]. Recently, the significance of dissecting the extracellular/intracellular contribution to Cr(VI) reduction under aerobic and anaerobic conditions has been acknowledged. The ability of bacterial Cr(VI) reduction under aerobic and anaerobic conditions has been simultaneously investigated in *Sporosarcina saromensis* W5 and *Exiguobacterium* sp. PY14, respectively [13,14]. It is reported that the ability of Cr(VI) reduction under anaerobic conditions is greater than that of aerobic in the two bacterial species. Particularly, the intracellular components contributing to greater Cr(VI) reduction under aerobic and anaerobic conditions have been observed in *S. saromensis* W5 [14]. However, whether the intracellular components play a similar role in Cr(VI) reduction for other bacterial species remains unknown. Hence, it is essential to quantify the specific contributions of cellular components to reduce Cr(VI) under aerobic and anaerobic conditions to comprehensively understand the microbial Cr(VI)-reduction mechanism.

The present study aims to isolate a new bacterium to explore its resistance to Cr(VI), the rate of Cr(VI) reduction, and the extracellular and intracellular contributions to Cr(VI) reduction under aerobic and anaerobic conditions.

## 2. Materials and Methods

### 2.1. Screening and Identification of a Cr(VI)-Resistant Bacterium of Strain HGB10

A soil sample was collected from the area adjacent to Jilin Petrochemical, China, and transferred to the lab to isolate Cr(VI)-resistant bacteria without delay. A soil sample (1 g) was added to 100 mL of sterilized water to create serial-dilution suspensions ranging from 10^−3^–10^−6^. The resultant suspension culture was spread onto LB agar plates containing 5 mg L^−1^ Cr(VI) and aerobically incubated at 30 °C for two days. Isolates capable of growing on the plate were selected to further screen higher Cr(VI) resistance in an LB liquid medium containing 20–100 mg L^−1^ under aerobic/anaerobic conditions. Single colonies from each candidate were randomly picked, among which an isolate designated as HGB10 was ultimately chosen due to its rapid growth.

The identification of strain HGB10 was conducted based on 16S rRNA phylogeny characteristics. A single colony of HGB10 strain from a freshly streaked LB-agar plate was inoculated in an LB culture for a genomic DNA extraction kit (TaKaRa Biotechnology Company Limited, Dalian, China). The methods for bacterial species identification, including PCR-amplified 16S rRNA by bacterial universal primer and comparison with the NCBI database using BLAST, were referred to in our previous work [15].

### 2.2. Cultivation

In aerobic experiments, the HGB10 strain was routinely cultivated in a 250 mL flask containing 100 mL of working-volume LB medium at 30 °C, shaking incubators with 160 rpm. In the case of anaerobic experiments, cell cultivation was performed in a wide-mouth bottle with strainless, sealed plastic caps featuring two perforated holes and silicone washers to maintain the airtightness in the anaerobic bottle. At the onset of the anaerobic experiment, the autoclaved anaerobic bottle with the medium was flushed with nitrogen gasses for 20 min to expel the dissolved oxygen in the medium and simultaneously to fill nitrogen gasses into the aluminum foil bag connected with the cap of the anaerobic bottles. In this manner, the stored nitrogen gasses in the aluminum foil bag could maintain the anaerobic atmosphere in the anaerobic bottles when the intake valve from the nitrogen cylinder was closed. The anaerobic bottles were placed in the incubator at 30 °C for static cultivation during the experimental process. As such, the dissolved oxygen in the anaerobic bottle can keep a lower than 0.2 mg L^−1^ [16].

### 2.3. Minimum Inhibitory Concentration (MIC) Test for Cr(VI)

The aerobic/anaerobic MIC values for Cr(VI) against strain HGB10 were determined using LB broth cultures. The aerobic/anaerobic cell cultivation methods were the same as those described in the cultivation section. The MIC testing approach had been described in our prior study [15]. Briefly, the 10,000 mg L^−1^ stock solutions of K_2_Cr_2_O_7_ were prepared using analytical-grade chemicals and sterilized through filtration. Subsequently, a series of Cr(VI) concentrations were prepared in LB cultures initially by two-fold dilution. Finally, strain HGB10 was aerobically/anaerobically inculcated into each culture with different Cr(VI) concentrations, and its OD_600_ was measured from 0 h to 120 h cultivation at a specific time interval. In all the subsequent experiments, the Cr(VI) concentration was set at 20 mg L^−1^. 

### 2.4. Determining the Cr(VI) Reduction Rate and Extracellular and Intracellular Contribution under Aerobic and Anaerobic Conditions

The total Cr was determined using inductively coupled plasma optical emission spectroscopy (ICP-OES, Avio 200, PerkinElmer, Waltham, MA, USA). Cr(VI) was determined by the 1,5-diphenylcarbazide spectrophotometric method at 540 nm [17]. The Cr(III) was obtained by calculating the total Cr minus Cr(VI). In the experiments for determining aerobic/anaerobic Cr(VI)-reduction rate, the culture of the HGB10 strain, after being aerobically/anaerobically cultured for 96 h and 120 h in the presence/absence of Cr(VI), was centrifuged at 12,000× *g* for 10 min at four °C. The resulting cell-free suspension directly determined the remaining Cr(VI) after aerobic/anaerobic Cr(VI) reduction. Then, the Cr(VI)-reduction rate was calculated according to the following formula:

Cr(VI)-reduction rate % = (C_0_ − C_t_)/C_0_ × 100% (1)

where C_0_ (mg L^−1^): initial concentration of Cr(VI);

C_t_ (mg L^−1^): concentration of Cr(VI) at t time (finishing time).

Independent Cr(VI)-reduction experiments were carried out to dissect extracellular and intracellular contributions to Cr(VI) reduction under aerobic and anaerobic conditions. The bacterial culture was centrifuged after 96 h and 120 h of aerobic/anaerobic cultivation, with or without Cr(VI). The resulting cell-free supernatants and cell pellets determined the extracellular/intracellular Cr(VI) content. The extracellular Cr(VI) contents are distributed in the soluble extracellular polymeric substances (S-EPS) and the binding EPS (B-EPS) [18]. The filtered cell-free supernatants passing through a 0.22 μm membrane were determined for S-EPS Cr(VI). The phosphate-buffered-saline-washed cell pellets were added to EDTA and centrifuged. The resulting supernatants were determined for B-EPS Cr(VI). Regarding the intracellular Cr(VI) content, the washed cell pellets were disrupted by ultrasonic waves. Subsequently, the aqueous and the remaining part, which were digested using acid, were employed to determine the cytoplasm and membrane of the total Cr and Cr(VI), respectively [19]. Eventually, the overall extracellular and intracellular contribution to Cr(VI) reduction was calculated based on the extracellular and intracellular total Cr and Cr(VI) content.

### 2.5. Scanning Electron Microscope (SEM)

Four types of cells of strain HGB10 were prepared after 96 h and 120 h cultivation with or without supplementation Cr(VI) under aerobic and anaerobic conditions. The aerobic/anaerobic cells without Cr(VI) were used as controls for aerobic and anaerobic Cr(VI) stress, respectively. The various resulting cell suspensions obtained, as described in the cultivation section, were centrifuged at 12,000× *g* for 10 min at four °C. The cell pellets for SEM analysis were fixed by glutaraldehyde and dehydrated through a series of ethanol [20,21].

### 2.6. Data Analysis

Experiments in this study were performed in triplicate, and the data were presented as the mean ± SD. Statistical analyses were conducted using SPSS (version 21.0, IBM, Chicago, IL, USA). The statistical differences in the extracellular/intracellular contribution to Cr(VI) between aerobic and anaerobic conditions were determined by applying independent samples *t*-test (*** *p* < 0.001; * *p* < 0.05).

## 3. Results and Discussion

### 3.1. Isolation Pseudomonas sp. HGB10

**Figure 1:**
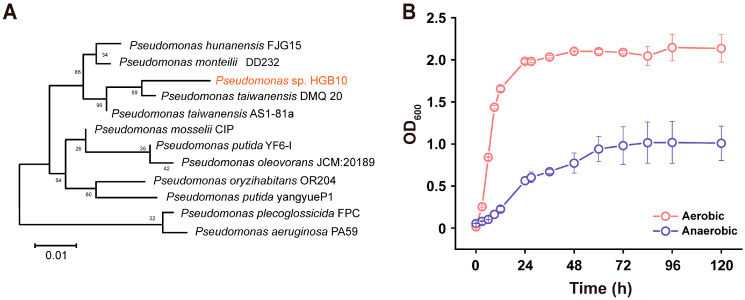
Phylogenetic tree of *Pseudomonas* sp. HGB10 and aerobic/anaerobic growth characterizations. (**A**) The phylogenetic tree was established based on the 16S rRNA gene sequences; (**B**) the growth characterizations under aerobic and anaerobic conditions.

Among the six isolated candidates capable of growing in the presence of Cr(VI) ranging from 20 to 100 mg L^−1^, one strain designated as HGB10 was selected for further investigation due to its elevated growth rates under aerobic and anaerobic conditions. After one day of incubation at 30 °C on LB agar medium, strain HGB10 produced 2–3 mm milky white, smooth, and moist colonies. The 16S rRNA gene sequence (1385 bp) of strain HGB10 (NCBI GeneBank accession OL307678.1) was obtained. The BLAST search results based on NCBI databases indicated that strain HGB10 shared 99.56% identity to *Pseudomonas taiwanensis* strain DMQ20 (Figure 1A). Conventionally, an identity of 16S rRNA sequence over 97% represents the same bacterial species [22]. Despite this, strain HGB10 was provisionally identified as *Pseudomonas* sp. HGB10, considering that its physiological and biochemical characteristics have not been tested.

### 3.2. The Growth Characteristics of Pseudomonas sp. HGB10 under Aerobic/Anaerobic Conditions

*Pseudomonas* sp. HGB10 can grow under both aerobic and anaerobic conditions. Notably, the cells exhibited a faster growth rate under aerobic conditions as compared to anaerobic conditions (Figure 1B). At their respective stable periods, the maximum OD_600_ of aerobic cells (2.2) was approximately twice that of the anaerobic cells (1.0). Members of the genus *Pseudomonas* are known for their adaptability to fluctuating environmental circumstances due to their versatile metabolic capabilities [23]. Regarding oxygen availability, *Pseudomonas* spp. can inhabit diverse oxygen conditions, encompassing aerobic and anaerobic conditions [24]. The capability of strain HGB10 to grow and sustain aerobically/anaerobically makes it an ideal experimental model for investigating the Cr(VI) reduction under aerobic and anaerobic conditions in our subsequent studies.

### 3.3. Resistance to Cr(VI) of Pseudomonas sp. HGB10 under Aerobic/Anaerobic Conditions

**Figure 2:**
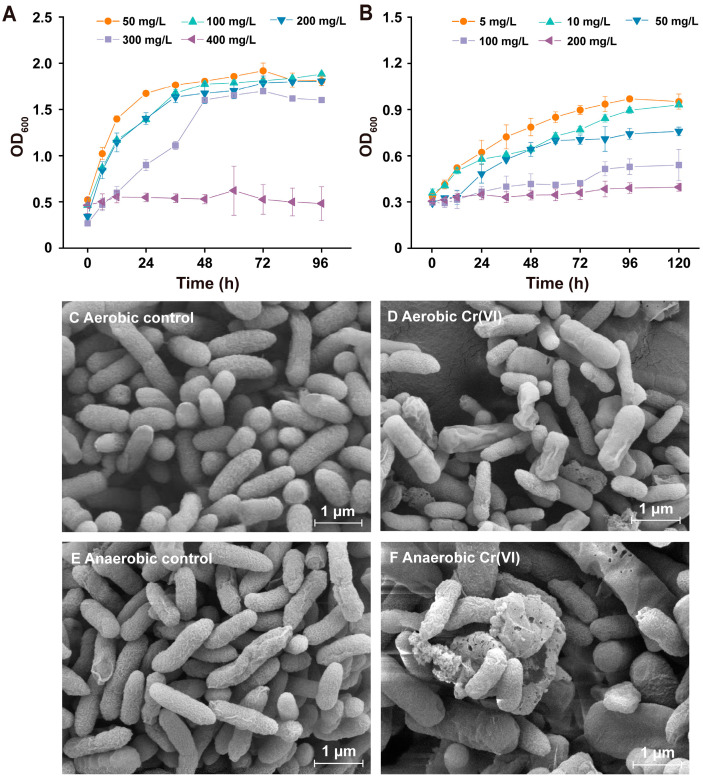
The growth of *Pseudomonas* sp. HGB10 in the absence and presence of different concentrations of Cr(VI) in LB media under aerobic and anaerobic conditions. (**A**) Aerobic conditions; (**B**) anaerobic conditions. (**C**–**F**) Scanning electron micrographs of *Pseudomonas* sp. HGB10 were exposed to 20 mg L^−1^ concentrations of Cr(VI).

Strain HGB10 demonstrated superior aerobic Cr(VI)-resistance compared to anaerobic resistance. The aerobic MIC at 400 mg L^−1^ was twice as high as that of the anaerobic at 200 mg L^−1^, indicating that the toxicity of Cr(VI) under anaerobic conditions is more pronounced than that under aerobic conditions (Figure 2A,B). The SEM images provided detailed information regarding the morphological alterations in aerobic/anaerobic cells with/without Cr(VI) stress (Figure 2C–F). The observable distinction was that there were more cell aggregations and a rougher cell wall under anaerobic conditions than those under aerobic conditions when the two types of cells were subjected to the same concentration of Cr(VI) stress (20 mg L^−1^) in contrast to their corresponding controls, respectively. Similarly, in *E. coli*, increased toxicity of Cu(II) has been observed under anaerobic conditions in contrast to aerobic conditions, as cells of *E. coli* modulate their Cu(II) efflux system in response to Cu and oxygen availability [25]. We infer that strain HGB10 likely adopts a comparable strategy to overcome the Cr(VI) stress. However, as no studies have compared the toxicity of Cr(VI) under aerobic and anaerobic conditions from the perspective of transcriptomics, further investigations are required to verify whether strain HGB10 transcriptional regulation responses to Cr(VI) and anaerobic conditions are required.

### 3.4. The Anaerobic Cr(VI)-Reducing Rate of Pseudomonas sp. HGB10 Preponderates over the Aerobic Counterpart

**Figure 3:**
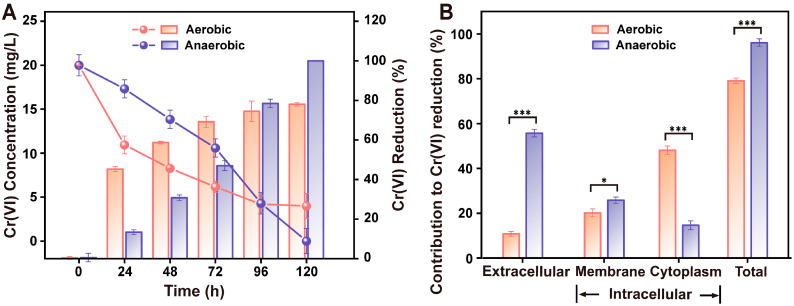
*Pseudomonas* sp. HGB10 Cr(VI)-reducing rate and extracellular/intracellular contribution to Cr(VI) reduction under aerobic and anaerobic conditions. (**A**) The Cr(VI)-reducing rate was determined when the initial concentration of Cr(VI) was 20 mg L^−1^; (**B**) the extracellular/intracellular contribution was calculated based on the remaining content of the total Cr, Cr(VI), and Cr(III) (mg per gram dry cell weight) after aerobic/anaerobic Cr(VI) reduction. The significance of differences among charts (**B**) according to samples *t*-test (***, *p* < 0.001; *, *p* < 0.05).

Both aerobic- and anaerobic-growing cells can reduce Cr(VI). Nevertheless, these two types of cells demonstrated distinct Cr(VI)-reducing characteristics throughout the entire reduction process (Figure 3A). The anaerobic Cr(VI)-reducing capacity surpassed that of the aerobic counterpart; the Cr(VI)-reducing rate of anaerobic cells reached 100% at 120 h, while the Cr(VI)-reducing rate of aerobic cells reached equilibrium at 96 h (75%). The more potent Cr(VI)-reduction ability in anaerobic/anaerobic cells might be attributed to the higher redox potential of Cr(VI) (1.33 V) compared with oxygen (1.23 V) [26]. In this respect, a similar phenomenon is witnessed in facultative anaerobic/anaerobic bacteria of *S. saromensis* W5 and *Exiguobacterium* sp. PY14 [13,14]. These two species represent the only two studies comparing aerobic/anaerobic Cr(VI)-reduction ability using a bacterial species.

It is also interesting to note that the relatively lower anaerobic MIC to Cr(VI) (200 mg L^−1^) compared to the aerobic one (400 mg L^−1^) did not influence the capacity of anaerobic Cr(VI) reduction. On the contrary, the maximum anaerobic Cr(VI)-reducing rate was 25% higher than that of the aerobic counterpart (Figure 3A). Our study corroborates that Cr(VI) resistance and reduction are not necessarily interrelated but independent [27]. In fact, some bacteria that are not resistant to Cr(VI) can still reduce Cr(VI) to Cr(III), but their growth is notably inhibited at higher concentrations of Cr(VI) [11,28]. Therefore, the bacterial characteristic utilized for effective Cr(VI) bioremediation depends on the combination of high resistance and the Cr(VI)-reductive ability [29].

### 3.5. The Extracellular/Intracellular Contribution to Cr(VI) Reduction under Aerobic/Anaerobic Condition

To uncover the reason behind the superior Cr(VI)-reducing ability of *Pseudomonas* sp. HGB10 under anaerobic conditions compared to the aerobic counterpart, we quantified the extracellular and intracellular contribution to Cr(VI) reduction under aerobic and anaerobic conditions and carried out a corresponding statistical *t*-test (Figure 3B). Specifically, the maximum aerobic (79.05%) was nearly in line with the Cr(VI)-reducing rate under aerobic (75%) by directly measuring the remaining Cr(VI) in the solution. Similarly, the maximum anaerobic (96.09%) was very close to the direct measurement result (100%), although there were minor relative errors for both aerobic (4.05%) and anaerobic cells (3.91%) due to multiple measurements of different fractions of Cr(VI). Regarding extracellular Cr(VI) reduction, the anaerobic extracellular contribution (55.71%) was significantly much higher than that of the aerobic one (10.76%) (*p* < 0.001). In contrast, the aerobic intracellular Cr(VI) reduction (68.29%) was significantly higher than that of the anaerobic one (40.38%) (*p* < 0.05). Overall, the anaerobic Cr(VI) reduction was significantly higher by 17.09% compared to the aerobic one (*p* < 0.05).

Bacterial Cr(VI) reduction is a combined process involving both intracellular and extracellular roles [30,31]. Multiple reductases, non-reductases like glutathione and ascorbate, and various electron-transfer shuttles, for instance, flavins and ubiquinone, are known to be responsible for the Cr(VI) reduction [11,28,32]. Thus, quantifying simultaneous extracellular and intracellular contributions to Cr(VI) reduction is crucial for understanding bacterial Cr(VI)-reduction mechanisms. However, to date, only one published work has simultaneously compared intracellular and extracellular contributions to Cr(VI) reduction under aerobic and anaerobic conditions. In their study, the intracellular component of *S. saromensis* W5 contributes to Cr(VI) reduction under aerobic and anaerobic conditions; no obvious extracellular Cr(VI) reduction occurs [14]. In our study, the intracellular component played the predominant role in Cr(VI) reduction under aerobic conditions, but anaerobic Cr(VI) reduction predominately occurred extracellularly. Based on the comparison between our *Pseudomonas* sp. HGB10 with *S. saromensis* W5, it can be concluded that the intracellular and extracellular roles in Cr(VI) reduction under aerobic and anaerobic conditions are species-dependent. Bacterial anaerobic Cr(VI) reductions have been reported to be predominant by three extracellular electron transport (EET) pathways: the cytochrome c, electron shuttles, and microbial nanowire [33,34]. Membrane-bound cytochrome c proteins (e.g., MtrC and OmcA) can directly transfer electrons to Cr(VI) [35]. Electron shuttles (such as riboflavin, Fe(III), and anthraquinone-2,6-disulfonate) can undergo reversible oxidation and reduction, thereby transferring electrons from microbes to Cr(VI) [36]. Additionally, microbial nanowires, composed of electrically conductive proteins, enable the transfer of electrons generated by bacteria to Cr(VI) [34]. In our preliminary study, although we found a distinctive phenomenon in *Pseudomonas* sp. HGB10, it should be examined which specific (EET) pathway or combination is responsible for anaerobic Cr(VI) reductions in strain HGB10 in our follow-up work. Despite this, it is interesting to note that our study emphasizes the possible implementation of bacterial Cr(VI) reduction under anaerobic conditions, as less low-dissolved oxygen demand means less energy-consuming in practical wastewater treatment.

## 4. Conclusions

The present study has demonstrated that the lower anaerobic minimum inhibitory concentration Cr(VI) does not imply a lower anaerobic Cr(VI)-reduction rate for an isolated Cr(VI)-reducing bacterium of *Pseudomonas* sp. HGB10. In fact, for the initial Cr(VI) concentration of 20 mg L^−1^, the maximum anaerobic Cr(VI)-reducing rate reached 100%, while the aerobic counterpart was only 75%, even though the value of aerobic MIC (400 mg L^−1^) is twice of the anaerobic (200 mg L^−1^). Additionally, this study has clarified the extracellular and intracellular contributions to Cr(VI) reduction under aerobic and anaerobic conditions. The extracellular reductions significantly contribute to anaerobic Cr(VI) reduction, while the intracellular components significantly contribute to aerobic Cr(VI) reduction. Thus, oxygen condition plays a vital role in contributing to bacterial Cr(VI) reduction. Taken together, findings from this study advance the understanding of the microbial Cr(VI)-reduction mechanism and may facilitate the application of bacterial reduction of Cr(VI) in more anaerobic environments.

## Data Availability

The original contributions presented in the study are included in the article, further inquiries can be directed to the corresponding author.
